# Comment: In Response to “Megaloblastic Anemia with Ring Sideroblasts is not Always Myelodysplastic Syndrome”

**DOI:** 10.4274/tjh.2016.0466

**Published:** 2017-03-01

**Authors:** Smeeta Gajendra

**Affiliations:** 1 Medanta-The Medicity, Department of Pathology and Laboratory Medicine, Gurgaon, India

**Keywords:** Refractory anemia with ring sideroblasts, RARS with thrombocytosis, Myelodysplastic syndrome/myeloproliferative neoplasm with ring sideroblasts and thrombocytosis

## TO THE EDITOR,

I read the letter “Megaloblastic Anemia with Ring Sideroblasts is not Always Myelodysplastic Syndrome” by Narang et al., recently published in this journal [1]. The manuscript is well written with a description of a very informative case of megaloblastic anemia with ring sideroblasts in a young female of 18 years old. Ring sideroblasts are associated with abnormal expression of several genes of heme synthesis or mitochondrial iron processing [2]. After exclusion of non-neoplastic causes of ring sideroblasts such as congenital/hereditary sideroblastic anemia and acquired reversible sideroblastic anemia (drugs, toxins, or nutritional deficiency), myelodysplastic syndrome (MDS) can be strongly suspected, particularly in elderly patients. The presence of ring sideroblasts alone is not sufficient for a diagnosis MDS; the presence of refractory cytopenia(s) is a prerequisite. Refractoriness can only be established after exclusion of secondary causes, most importantly nutritional deficiencies. After that, a complete evaluation of the erythroid, myeloid, and megakaryocytic lineages of bone marrow is essential. At least 15% ring sideroblasts are required for the diagnosis of MDS with ring sideroblasts (MDS-RS) in cases lacking mutations in the spliceosome gene *SF3B1*. *SF3B1* mutations are found in 60%-80% of patients with refractory anemia with ring sideroblasts (RARS) or RARS with thrombocytosis (RARS-T) and are associated with favorable prognosis [3]. In the recent World Health Organization (WHO) 2016 classification, cases with ring sideroblasts and multilineage dysplasia without excess blasts or isolated del (5q) abnormality are categorized as MDS-RS. Recent studies have shown that the percentage of ring sideroblasts in MDS is not prognostically important. Thus, in the revised WHO classification, a diagnosis of MDS-RS may be made even in the presence of only 5% of ring sideroblasts in cases with *SF3B1* mutation. MDS-RS cases will be subdivided into cases with single lineage dysplasia (previously classified as RARS) and cases with multilineage dysplasia (previously classified as refractory cytopenia with multilineage dysplasia). Furthermore, RARS-T has been accepted as an entity and termed MDS/myeloproliferative neoplasm (MPN) with ring sideroblasts and thrombocytosis (MDS/MPN-RS-T) in the 2016 classification. Unlike MDS-RS, the number of ring sideroblasts required for a diagnosis of MDS/MPN-RS-T is 15%, irrespective of the presence or absence of a *SF3B1* mutation [4]. As described in the case of Narang et al., in a young female of 18 years old without a history of persistent refractory cytopenia(s), a diagnosis of MDS can only be established after exclusion of secondary causes such as nutritional deficiencies [1]. An adequate trial with hematinics (vitamin B12, folic acid, and pyridoxine) is needed in such cases. After exclusion of secondary causes, if cytopenia(s) still persists, a repeat bone marrow examination with cytogenetic and molecular studies can be considered to establish the diagnosis of a clonal hematopoietic disease such as MDS or MDS/MPN.
